# A Seed-Endophytic *Bacillus safensis* Strain With Antimicrobial Activity Has Genes for Novel Bacteriocin-Like Antimicrobial Peptides

**DOI:** 10.3389/fmicb.2021.734216

**Published:** 2021-09-27

**Authors:** Jeanne Romero-Severson, Thomas E. Moran, Donna G. Shrader, Francisco R. Fields, Susan Pandey-Joshi, Clayton L. Thomas, Emily C. Palmer, Joshua D. Shrout, Michael E. Pfrender, Shaun W. Lee

**Affiliations:** ^1^Department of Biological Sciences, University of Notre Dame, Notre Dame, IN, United States; ^2^Department of Civil and Environmental Engineering, University of Notre Dame, Notre Dame, IN, United States

**Keywords:** bacteriocins, *Bacillus*, endophyte, commensalism, plant microbiome

## Abstract

Bacteriocins are a highly diverse group of antimicrobial peptides that have been identified in a wide range of commensal and probiotic organisms, especially those resident in host microbiomes. Rising antibiotic resistance have fueled renewed research into new drug scaffolds such as antimicrobial peptides for use in therapeutics. In this investigation, we examined mung bean seeds for endophytes possessing activity against human and plant pathogens. We isolated a novel strain of *Bacillus safensis*, from the contents of surface-sterilized mung bean seed, which we termed *B. safensis C3*. Genome sequencing of C3 identified three distinct biosynthetic systems that produce bacteriocin-based peptides. C3 exhibited antibacterial activity against *Escherichia coli, Xanthomonas axonopodis*, and *Pseudomonas syringae*. Robust antimicrobial activity of *B. safensis* C3 was observed when C3 was co-cultured with *Bacillus subtilis*. Using the cell-free supernatant of C3 and cation exchange chromatography, we enriched a product that retained antimicrobial activity against *B. subtilis.* The peptide was found to be approximately 3.3 kDa in size by mass spectrometry, and resistant to proteolysis by Carboxypeptidase Y and Endoproteinase GluC, suggesting that it is a modified variant of an AS-48 like bacteriocin. Our findings open new avenues into further development of novel bacteriocin-based scaffolds for therapeutic development, as well as further investigations into how our discoveries of bacteriocin-producing plant commensal microorganisms may have the potential for an immediate impact on the safety of food supplies.

## Introduction

Antimicrobial resistance (AMR) is a current global issue that is likely to accelerate in concern in the next decade (CDC, 2019; WHO, 2021). One study has projected that by 2050, AMR infections will result in upward of 10 million deaths annually (O’Neill, 2019). While antibiotic misuse is the major driver of AMR, the absence of research into effective antibiotic development has contributed significantly to this crisis (Alanis, 2005). To mitigate the approaching catastrophe, global collaboration, and extensive clinical research into new therapies will be needed. Novel scaffolds for antibiotics are especially needed given the high rate of resistance to existing drug templates. In this aspect, growing attention has been allocated one class of therapeutics with promise known as antimicrobial peptides (AMPs) (Ageitos et al., 2017).

While AMPs can be found in many organisms, bacteriocins are natural ribosomally-synthesized AMPs produced by bacteria. These products have broad applications as food preservatives, clinical therapeutics, and agricultural additives (O’Connor et al., 2020). Generally, bacteriocins are useful because they are potently effective at nanomolar concentrations, molecularly stable over a wide range of pHs and temperatures (Johnson et al., 2018), considered to have low toxicity (Maher and McClean, 2006; Castiglione et al., 2007), and less likely to lead to drug resistance (de Freire Bastos et al., 2015; Draper et al., 2015; Geldart and Kaznessis, 2017). Some of these peptides are considered narrow spectrum, killing closely related species, while others have broad spectrum activity (Cotter et al., 2013). While there are many different mechanisms of action described, in general, bacteriocins function by targeting the surface of the bacterial cell, resulting in membrane permeabilization (Cotter et al., 2013; Lv et al., 2014; Ong et al., 2014). For example, Enterocin AS-48 it thought to insert itself into the lipid bilayer and form pores, rendering the target cell non-viable as the membrane loses integrity (José et al., 2014). In contrast, Nisin and other lantibiotics bind to lipid II, which blocks cell wall synthesis (Bierbaum and Sahl, 2009).

In nature, these AMPs may serve to benefit the producer species by reducing competition within an environmental niche, or by preventing harmful pathogens from destroying their organismal host (Hassan et al., 2012; Zschüttig et al., 2012; Simons et al., 2020). In particular, microbial endophytes that live as symbiotic organisms with plants have been investigated for their role in microbial competition and maintenance of host niche. Previous studies have shown that endophytes can exhibit antimicrobial activity and may contribute to pathogen resistance (Singh et al., 2017; Fadiji and Babalola, 2020). Through their beneficial roles, endophytes present a natural means to aid in protecting vegetative crops for consumption. Recent outbreaks of *Salmonella* in the United States and Canada have been connected with the consumption of sprouts from the mung bean plant, *Vigna radiata* (Yang et al., 2013). There are limited studies on mung bean endophytes and their potential roles in host-microbe protective mutualism, especially as it pertains to the specific role of antimicrobial products produced by potential resident microbes of *V. radiata.*

The bacterium species *Bacillus safensis* is a member of the ubiquitous, Gram-positive, endospore-forming genus *Bacillus*. Originally *B. safensis* was isolated from the surfaces of spacecraft within assembly facilities in California and Florida (Satomi et al., 2006). Since its identification, it has been isolated from a wide range of environments, including surface soil (Ishag et al., 2016), oil fields (Rekik et al., 2019), and plant microbiomes (Wahla et al., 2019). Strains of *B. safensis* have demonstrated antifungal activity (Mayer and Kronstad, 2017), degradation of petroleum pollutants (Wu et al., 2019), and the promotion of plant growth (Sarkar et al., 2018). In biotechnology research, *Bacillus* species are significant due to their ability to produce structurally diverse arrays of secondary metabolites, including antimicrobial compounds (Baindara et al., 2013; Sumi et al., 2015; Fira et al., 2018).

In this study, we sought to isolate potential endophytes present within the seeds of the mung bean plant, *V. radiata*. We hypothesized that the bacteria resident in plant sources could be a potential source of novel bacteriocin discovery. We report here the isolation of a unique, highly-motile, Gram-positive bacterium which we identify, based on the whole genome sequence, as a new strain of *B. safensis* named C3. To demonstrate a non-pathogenic role for *B. safensis* C3, we studied the uptake of *B. safensis* by *V. radiata* during imbibition and the bacteria colonize the tissue of sprouted seeds. We provide sequence data and functional studies to demonstrate that this strain of *B. safensis* contains genes encoding several putative bacteriocin-like AMPs. The bacteriocins produced by C3 could play a significant role in preventing bacterial colonization by non-commensal microorganisms during seed imbibition and sprouting.

## Materials and Methods

### Seed Sources

We designated as seed lot 1 (SL1) mung bean [*V. radiata* (L.) R. Wilczek] seed purchased from Totally Tomatoes (Randolph, WI, seed lot year 2007, item number 04420N). Seeds were stored in the original packet under cool and dry conditions. Over 99% of the seed germinated in 2008 and in all tests done in 2011 and 2012. Seed lot 2 (SL2) was purchased from a local retailer (Shelton Farms, Niles, MI) in 2012. The provenance and age of this lot is unknown. However, SL2 also germinated at more than 99% in preliminary tests.

### Surface Sterilization, Intact Seed Plating, and Seed Grinding Procedures

We surfaced sterilized dry, intact mung bean seed in 0.66% sodium hypochlorite for 10 min in a sterile petri dish, to allow full surface contact of the sodium hypochlorite to the beans, rinsed in a 50 mL conical tube with sterile distilled H_2_O, then submerged the seed in a new sterile petri dish in a 70% ethanol solution for 10 min. The ethanol solution was aspirated with a vacuum flask and the residual ethanol evaporated in a laminar flow hood. We tested each surface sterilized seed sample with control test plates consisting of 8–10 surface sterilized seeds placed in each of four plates of LB agar. We observed test plates after 48 h at 37°C or after 8 days at 20°C. Seeds destined to be plated intact were then plated under a laminar flow hood until plating. Otherwise, seeds were ground using a SPEX CertiPrep Freezer/Mill 6850 (Metuchen, NJ). This device immerses tubes containing the sample into liquid nitrogen and pulverizes the contents using a steel rod. Tubes (Certi-prep model number 6751), steel rods and caps were soaked in 70% ethanol for 10 min prior to grinding and the ethanol allowed to evaporate. Immediately after grinding, the bean seed powder was transferred to sterile Whirl-Paks (Nasco, Fort Atkinson, WI) and stored at –80°C until testing. Bean powder (∼1 g) was tapped into 30 mL of lysogeny broth (LB, EMD chemicals, Gibbstown, NJ) or Todd Hewitt (TH) broth (Neogen Corporation, Lansing, MI), grown overnight at 37°C shaking at 225 rpm. These cultures were then plated undiluted, 1:10 and 1:100 onto the same nutrients in agar and then incubated overnight at 37°C. Following the initial isolation of bacterial endophytes from mung bean seeds, all seed sterilization, seed plating and seed grinding procedures were done in the Plant/Tree Genomics Core Facility at University of Notre Dame (Notre Dame, IN), to reduce the risk of contamination by other microbes.

### Isolation and Identification of Seed Bacterial Endophytes

Three bacterial colonies were selected from the 1:100 dilution TH plates. Upon observing the plates, it was determined that there were only three unique colony morphologies on the plates, thus one colony of each unique morphological type was selected for 16S ribosomal RNA gene sequencing. DNA was amplified directly from the three selected colonies using universal primers for the 16S ribosomal subunit (see Supplementary Materials for primer sequences; Eden et al., 1991; Weisburg et al., 1991). The PCR reaction was carried out by adding a colony to a 50 μL reaction volume: 5 μL of 20 mM MgCl_2_, 5 μL of Standard 10X Taq Reaction Buffer (New England BioLabs, Ipswich, MA), 2 μM dNTPs, 0.2 pM/μL each forward and reverse primer and 0.625 μL Takara Taq polymerase (Clontech Laboratories, Inc., Mountain View, CA), at the following conditions: 2 min at 94°C, 35 cycles of 35 s at 94°C, 45 s at 55°C, and 2 min at 72°C, with a final extension for 2 min at 72°C. PCR products were sequenced using the Sanger sequencing method on an Applied Biosystems 3730xl DNA Analyzer (Foster City, CA). The genera for each of our resulting 16S ribosomal subunit sequences were identified using the BLASTn tool found on the NCBI webserver. The bacterial strain isolate selected for further study was identified as *B. safensis* C3. Throughout the study, C3 was grown in the following nutrient media: LB, TH broth, or nutrient broth (Sigma-Aldrich, St. Louis, MO). The C3 isolate was grown at 37°C either in liquid cultures or on 1.5% agar plates.

### Reisolation of C3

We reisolated C3 from our original seed lot after our initial discovery. For reisolation of C3, we ground an additional four sets of 100 surface-sterilized seeds, plus intact seed controls, from our original seed lot (SL1) and four sets from another seed lot (SL2, different supplier) for additional testing. In a subsequent experiment we surface sterilized and ground twenty-two seeds from SL1, incubated and plated as before, but only in TH media.

### Colony PCR

We assumed C3 specificity based on BLASTn searches and chose a unique genetic region that was not found in any other nr NCBI genome databases. Designed primers to target a sequence with the gene of a putative Glyoxylase family protein according to RAST homology annotation, with no similar nucleotide homology hits from BLASTn using the NCBI nr database. This amplicon has an expected size of 441 bp and produces a single band at *T*_*m*_ 57–65°C. Primers for this product were designed with restriction sites for future subcloning (see Supplementary Materials for primer sequences). Colony PCR was performed by adding a colony to a 50 μL reaction volume as prepared above for 16S ribosomal subunit PCR. The reaction occurred at the following conditions: 5 min at 94°C, 35 cycles of 30 s at 94°C, 30 s at 60°C, and 70°C, with a final extension for 30 min at 70°C. All colony PCR tests included a positive control from the original C3 strain and a negative control with water as the template.

### Determination of Growth Dynamics

To measure growth dynamics, C3 was grown in LB until the OD_600_ reached a value of 0.8, then the liquid culture was diluted 1:1,000 into fresh sterile LB (Mayer and Kronstad, 2017). Cultures were incubated in a 96-well plate at 37°C while shaking using a Synergy H1 plate reader (Biotek, Winooski, VT). The total medium volume was 200 μL per well. Bacterial growth was recorded by measuring the OD_600_ of the liquid media at 30 min intervals for 7 h and then at regular intervals the remaining 36 h. OD_600_ values were compared to the sterile media control.

### Biofilm Formation in Polystyrene Tubes

We assessed the capacity of C3 to form pellicles using polystyrene tubes (McQueary and Actis, 2011). An inoculum was prepared in LB and in TH broth from 18 h old agar colonies. The cultures were incubated overnight at 37°C without agitation. Biofilm formation at the air-liquid interface was observed visually followed by staining with crystal violet to confirm the formation of pellicles.

### Motility Assays

Swarming motility was assayed on LB medium solidified with 0.9% Noble agar (Difco Laboratories, Detroit, MI). Swarm plates were stab inoculated from broth cultures and incubated at 37°C. Some plate assays contained Syto59 bacterial-staining dye (Life Technologies, Grand Island, NY) and were inoculated with both C3 and *P. aeruginosa* (ATCC 15692) expressing green fluorescent protein (Gibiansky et al., 2010). Fluorescent images of swarm plates were acquired using a Carestream Multispectral FX (MSFX) imaging station (Carestream Health, Woodbridge, CT) using excitation and emission wavelengths for GFP and Nile Red were 480/535 and 540/600 nm, respectively (Morris et al., 2011).

### Detection of Antimicrobial Activity

We tested the antimicrobial activity of C3 through a series of disk diffusion assays (Bhunia et al., 1987). Using a hole punch, we made 6 mm disks from Whatman filter paper which were sterilized in the autoclave. Overnight cultures of our indicator strains (see below) were grown in nutrient broth incubated at 37°C while shaking (225 rpm). The overnight indicator strain liquid was added to molten soft agar (0.7%) at 1:100 v/v. The agar was gently mixed, poured into sterile petri dishes in 10 mL volumes, then allowed to cool for 30 mins. The C3 strain was prepared by mixing an isolated colony in LB liquid media and incubating overnight at 37°C shaking (225 rpm). The C3 liquid cultures were centrifuged at 1,791 × *g* for 10 min and 25 μL of the supernatant was removed and applied to the filter disks placed on the surface of soft nutrient agar plates previously inoculated with a competing species. Bacterial strains used in antimicrobial activity assays include *Escherichia coli* TOP10 (Life Technologies, Grand Island, NY), *Pseudomonas syringae* (kindly provided by R. Innes, Indiana University), *Xanthomonas axonopodis* pathovar Starr and Garces pathovar phaseoli (ATCC 9563), *Bacillus subtilis* (ATCC 6051). Plates were incubated at 37°C for 48–72 h followed by inspection of the zone of inhibition.

### Genome Sequencing

Genomic DNA was isolated with a DNeasy Blood and Tissue Kit following instructions provided by the manufacturer, Qiagen (Valencia, CA). We used 1 μg of genomic DNA harvested from C3 and prepared it for sequencing using the Illumina TruSeq DNA Sample Preparation V2 Kit (San Diego, CA) following manufacturer’s instructions. The sequencing was performed using an Illumina MiSeq System (San Diego, CA). Paired MiSeq read coverage ranged from 9.7X (C3) to up to 17X, and assemblies were performed with Velvet version 1.2.03 (Zerbino and Birney, 2008) using 31 as the *k*-mer value, 350 bp as the insert length, and automated coverage cutoffs (-exp_cov auto), as a specific value did not improve assembly. The MiSeq run and the assemblies were done at the Notre Dame Bioinformatics and Genomics Core (Notre Dame, IN). Genome annotation was performed using RASTtk (Brettin et al., 2015). This Whole Genome Shotgun project has been deposited at DDBJ/ENA/GenBank under the accession JAIFAE000000000. The version described in this manuscript is version JAIFAE010000000.

The genome sequences of C3 have been deposited to NCBI GenBank with the following hyperlink: https://www.ncbi.nlm.nih.gov/Traces/wgs/JAIFAE01?display=contigs.

### Reverse Transcriptase-PCR of C3 Bacteriocin Genes

RNA purification was performed using the RNeasy kit (Qiagen, Valencia, CA). C3 sonicate was used for all RNA preparations. RNA yield was quantified using nanodrop prior to proceeding with reverse transcription. Reverse transcription steps were performed using the QuantiTect Reverse Transcription kit per manufacturer instructions (Qiagen, Valencia, CA). Briefly, reverse-transcription PCR occurred at 42°C for 15 mins, then at 95°C for 3 min (see Supplementary Materials for primer sequences). Newly synthesized cDNA was stored at –20°C until used for PCR studies. Negative controls were performed for each experimental condition tested to eliminate false products from genomic contamination of samples.

### Bacteriocin Purification

From a plate, overnight cultures of *B. safensis* and *B. subtilis* were grown in LB and nutrient broth, respectively, shaking (225 rpm) at room temperature. A liter of sterile Murashige and Skoog Basal Medium (MS) was made with 1% soytone (w/v). In 1L Erlenmeyer flasks, 250 mL of MS (w/1% soytone) were aliquoted. Into each Erlenmeyer flask, 2.5 mL of overnight culture of both *B. safensis* and *B. subtilis* were added. Flasks were shaken (225 rpm) for 24 h at room temperature. Purification of bacteriocins from the cell supernatant was based on previously published methods with modifications (Gálvez et al., 1989; Song et al., 2014; Kaškonienë et al., 2017). Bacteria were then removed from the media by centrifugation at 10,000 × *g* for 10 min and the supernatant was filter sterilized using 0.22 μm bottle top vacuum filters (Corning Life Sciences, Tewksbury, MA). To the filtrate, 65% ammonium sulfate was added and left stirring overnight at 4°C. Precipitate was then enriched by centrifugation at 10,000 × *g* for 10 min and the supernatant was removed. Precipitate was resuspended in a total volume of approximately 20 mL of 20 mM H_2_NaPO_4_ (pH 6.2). The sample was dialyzed overnight to remove salt using 2,000 MWCO Slide-A-Lyzer Dialysis Cassettes (Thermo Scientific, Rockford, IL) against 20 mM H_2_NaPO_4_ (pH 6.2). Remaining precipitate was removed via centrifugation at 3,000 × *g* for 10 min. Once salt was diluted out, the sample was loaded onto a HiTrap SP HP column with the AKTA Pure (GE Healthcare, Chicago, IL). The peptide was eluted using 2 M NaCl 20 mM H_2_NaPO_4_ (pH 6.2) in 1 mL aliquots. Aliquots that represented individual peaks were combined and concentrated (3,000 × *g* for 60 min at 4°C) using 3K MWCO Macrosep Advance Centrifugal Devices (Pall Corporation, Port Washington, NY). The peptide was applied to a C-18 column. Protein was eluted using a 5–100% acetonitrile gradient. Peaks were pooled, vacuum dried, and resuspended in 20 mM H_2_NaPO_4_ (pH 6.2) buffer.

### Detection of Antimicrobial Activity of the Purified Bacteriocin Peptide

During the steps of bacteriocin induction and purification, samples were regularly tested for antimicrobial activity using a modification of the spot-on-lawn technique (Fujita et al., 2007). *Bacillus subtilis* was grown in nutrient broth overnight (225 rpm) at room temperature. Overnight culture was added in a 1:100 v/v to soft agar (0.7%), the agar was gently mixed and poured into sterile petri dishes in 10 mL volumes. After the media solidified, 5 μL of crude bacteriocin samples were spotted on the plate. Plates were incubated at 37°C overnight. Antimicrobial activity was determined by visually assessing the plates for a zone of inhibition.

### Mass Spectrometry

A 1 μL volume of an HPLC fraction containing peptide was added to 10 μL of a matrix solution (α-cyano-4-hydroxycinnamic acid). A 1 μL volume of the matrix-peptide media was then spotted and dried onto a target. Matrix-assisted laser desorption/ionization-time of flight (MALDI-TOF) MS analysis was performed using a Bruker UltrafleXtreme (Bruker, Billerica, MA). Instrument control and data processing software were Bruker FlexControl Version 3.4 and Bruker FlexAnalysis Version 3.4, respectively.

### Protein Sequencing

The HPLC purified fraction was loaded onto a ProSorb device (Applied Biosystems, Foster City, CA). The ProSorb membrane was then loaded onto a protein sequencing instrument, Shimadzu PPSQ-53A (Shimadzu, Columbia, MD) to carry out an Edman degradation sequencing method (Edman and Begg, 1967). The N-terminal amino acids were determined by comparing the retention times of the resulting PTH-amino acid from each Edman degradation cycle with the retention times of a standard mixture of 19 PTH-amino acids in a reference chromatogram.

### Proteolysis Assays

HPLC purified peptide was incubated in the presence of several enzymes in parallel reactions. The enzymes used were endoproteinase GluC (New England BioLabs, Ipswich, MA), leucine aminopeptidase (Sigma-Aldrich, St. Louis, MO), carboxypeptidase Y (Sigma-Aldrich, St. Louis, MO), trypsin (Thermo Fischer, Waltham, MA), and proteinase K (Promega, Madison, WI). The C3 peptide was incubated at 37°C for all reactions unless otherwise specified. The endoproteinase GluC reaction had a final concentration of 1X GluC reaction buffer (New England BioLabs, Ipswich, MA). The leucine aminopeptidase (Sigma-Aldrich, St. Louis, MO) reaction used a final concentration of 50 mM NaPO_4_ (pH 7.2). Cell grade trypsin/EDTA (0.25%) (Thermo Fischer, Waltham, MA) used deionized H_2_O for its reaction. Carboxypeptidase Y (Sigma-Aldrich, St. Louis, MO) reaction used a final concentration of 50 mM 2-[N-morpholino]ethanesulfonic acid (MES) pH 6.75, this reaction was carried out at 25°C. Enzyme concentrations were made at a ratio enzyme:substrate 1:50–1:100. Proteolytic reactions occurred over 4 h incubations or overnight. Peptide degradation was determined using MALDI-TOF as previously described. Recombinant SdrC, a purified protein was used as a control.

### Seed Inoculation of C3 Bacteria and Growth of *V. radiata* Seedlings

Seed inoculation studies were performed under sterile conditions using laminar flow hood assemblies as needed to avoid external contamination of seedlings. Seeds were first prepared for surface-sterilization with sodium hypochlorite and ethanol as previously described. The final ethanol rinse solution was aspirated completely with a vacuum flask and the residual ethanol evaporated in a laminar flow hood prior to seed imbibition. Whatman filter-paper soaked with a 1:2 diluted overnight culture of C3 in PBS was placed at the bottom of a 10 cm petri dish to which the seeds were placed. Three to four seeds were placed into each plate on pre-soaked filters and incubated in the dark for a period of approximately 24 h but monitored until seeds began to show visible indications of germination, at which time the seedlings were removed from the petri dish and transferred to a magenta box for growth of seedlings. Seedlings were placed in magenta growth boxes containing MS plant growth medium in agar (Carolina Biological Supply Company, Burlington, NC). *Vigna radiata* plants were placed directly under specific light conditions (14 h days light at 20°C) and grown for 7 days prior to processing, with young plants reaching an average height of 4–6 cm. Single plants were removed from boxes and placed on a TH agar to check for surface contamination. Middle plant stems lacking any external bacterial colony growth were transferred onto sterile plates for dissection. We chose the middle plant stem because that tissue is thinner and therefore better for imaging. Stems were dissected in half, lengthwise along the stem. The stems were washed with PBS and then incubated at room temperature with 4’,6-diamidino-2-phenylindole (DAPI; Cell Signaling Technology, Danvers, MA) nuclear stain for 2 h. The tissue was rinsed in PBS and then imaged on a Nikon Eclipse Ti-E microscope.

## Results

### Isolation and Phenotypic Characterization of C3

We observed three distinct colony morphology types on TH plates after incubating them overnight with ground mung bean seed tissue. Amplicon sequencing of 16S rRNA gene revealed three Gram-positive non-redundant bacterial genera, which we identified as *Enterococcus*, *Bacillus*, and *Staphylococcus*. Each of these was subsequently plated on a soft (0.8%) TH agar and incubated at 30°C to further examine colony morphology and motility. The colony morphology and surprising motility of one of these unique colony types (Colony 3), a *Bacillus* sp., immediately attracted interest. We subsequently focused our efforts on the characterization and whole genome sequencing of Colony 3 (C3).

**FIGURE 1:**
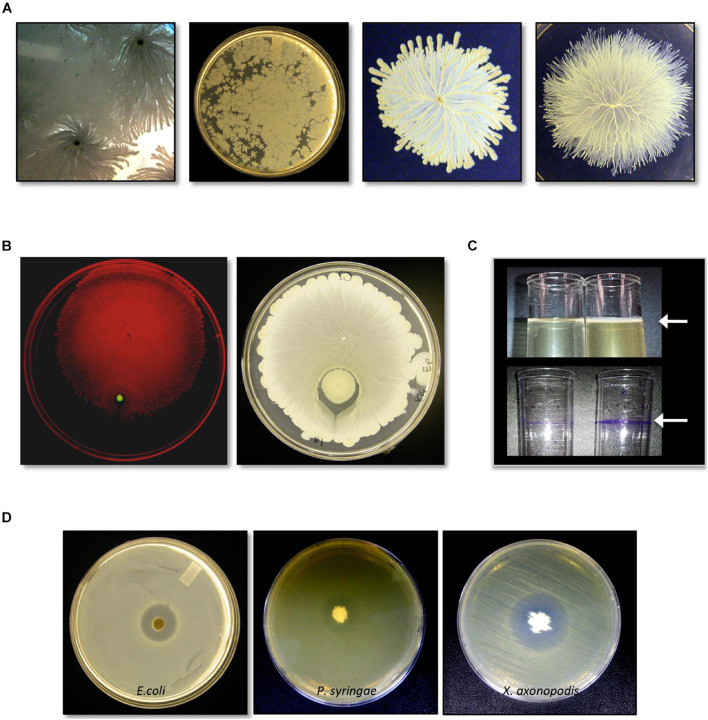
Identification and characterization of bacterial isolate *Bacillus safensis* C3 isolated from *Vigna radiata* seeds. **(A)** Growth and swarm motility behavior of C3. **(B)** C3 surrounds *P. aeruginosa* when inoculated as a co-culture on swarm agar plates. **(C)** When grown in culture broth, C3 forms a pellicle visible as a thin layer on top of the growing culture (top panel, arrow). Pellicles are visible following staining with crystal violet. **(D)** C3 extract displays antimicrobial activity against *Escherichia coli*, *Pseudomonas syringae*, and *Xanthomonas axonopodis* when grown in TH and nutrient agar plates.

C3 grew quickly in LB liquid culture at 37°C (Supplementary Figure 1) and was a rapid surface swarmer that exhibited a unique spreading pattern in which cell-dense tendrils formed within a monolayer of cells (Figure 1A). On a swarm assay plate, inoculated with C3 and *P. aeruginosa*, C3 swarming surrounded *P. aeruginosa* within 8 h at 37°C (Figure 1B). LB and TH broth cultures incubated overnight at 37°C without agitation formed a pellicle after 18 h in both LB and TH broth (Figure 1C). Crystal violet staining shows that biofilm extracellular matrix adhered to the polystyrene surface at the air-liquid interface.

We tested C3 antimicrobial activity using filter disks inoculated with an extract of C3 and placed on the surface of LB and TH agar plates previously spread with *E. coli*. C3 produced a clear inhibition zone only when grown on TH media (Figure 1D). C3 also inhibited the plant pathogens *X. axonopodis* and *P. syringae*. Notably, the strain of *X. axonopodis* used in these experiments (pathovar phaseoli) is a known pathogen of *V. radiata* (Hajri et al., 2009).

### Detection of Seed Surface Contamination and Reisolation of C3

Although we had ruled out incidental contamination of our isolates with other bacteria used in our laboratory facilities, recovery of an enterobacterium taxon in our preliminary experiments suggested the possibility of contamination during the isolation procedure. We therefore confirmed that no bacterial colonies could be detected on the surface of the mung bean seeds after 48 h at 37°C or after 8 days at 20°C (Supplementary Figure 2). In contrast, bacterial lawns typically appeared within 24–36 h in media in which untreated and unsterilized seeds were placed. Additionally, we repeated the isolation of C3 from additional seeds. Colony PCR of 12 unique colonies revealed one colony positive for the C3 specific amplicon (Supplementary Figure 3). These results suggest that *B. safensis* C3 is not packaged into every seed within a given seed lot.

### Genomic Characterization of C3

**FIGURE 2:**
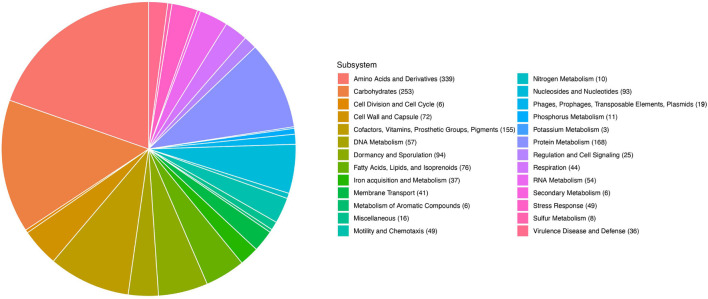
RAST annotation of the 2845 predicted genes in the C3 genome. Allocation of gene categories in the whole genome sequence of C3.

Illumina-based whole genome sequencing of C3 produced 3.7 MB of sequence in 32 contigs and a N50 of over 61 kb. RAST *in silico* annotations indicated that C3 genes dedicated to bacterial motility and chemotaxis (49 open reading frames, 2.69%), and dormancy and sporulation (94 open reading frames, 5.16%) are a prominent feature of this genome (Figure 2). A comparison of the C3 genome with the most complete genome of *B. safensis* strain JPL MERTA, using the genome comparison tool MUMmer4 yielded a 96.83% alignment overall (Marçais et al., 2018).

**FIGURE 3:**
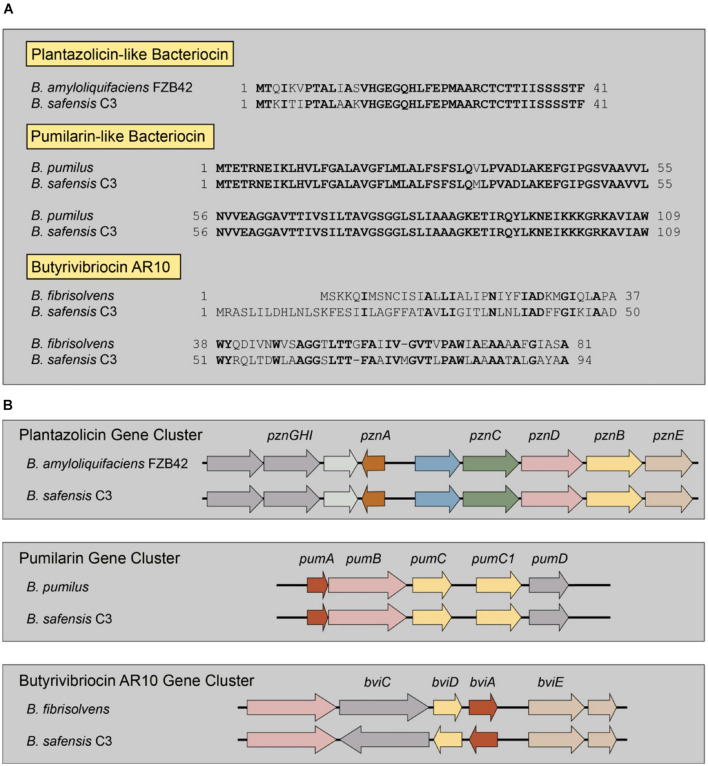
Identification of multiple bacteriocin genes present in the genome of C3. **(A)** Comparison of the amino acid sequences of known bacteriocins to those identified in the genome of C3. Residues in bold indicate identical amino acids. Butyrivibriocin AR10 from *Butyrivibrium fibrisolvens* and C3 possess highly similar C-terminal regions denoted by a double-glycine cleavage site (red italics). Clustal-based protein alignments are provided in supplement (Supplementary Figures 3, 4). **(B)** Presence and organization of bacteriocin biosynthetic gene clusters in the genome of C3.

From the genome sequence, we identified three distinct bacteriocin biosynthetic systems in the genome of the *B. safensis* C3 strain, using the bacteriocin-identification webserver BAGEL4 (van Heel et al., 2018). From BAGEL4, we were able to identify three putative bacteriocin genes and their associated biosynthetic gene clusters (Figure 3). The first putative bacteriocin found in C3 resembles a class of bacteriocins classified as linear azoline-containing peptides (LAPs). BLASTp analysis of the putative precursor peptide gene shows highest homology to the Plantazolicin bacteriocin compound from *Bacillus velezensis* FZB42 (previously classified as *Bacillus amyloliquifaciens*; Molohon et al., 2011; Scholz et al., 2011). Comparison of the protoxin gene product from the C3 strain and the Plantazolicin precursor revealed the greatest similarity at the C-terminus, with identical positioning of cysteine, threonine, and serine residues of the active bacteriocin product, which is similar, but not identical to the precursor peptide from *B. velezensis* FZB42 (Figure 3A). The second putative bacteriocin gene is nearly identical to the circular bacteriocin Pumilarin, previously identified from *B. pumilus* B4107 (van Heel et al., 2017). This putative C3 bacteriocin, which has been termed as Safencin (Fields et al., 2018), differs from Pumilarin by a single amino acid substitution (Figure 3A). The final bacteriocin gene found in C3 has homology to another circular bacteriocin, Butyrivibriocin AR10, from the bacterium *Butyrivibrium fibrisolvens* AR10 (Kalmokoff et al., 2003). Comparison of the Butyrivibriocin AR10 peptide sequence with the product from the C3 strain shows significantly less homology compared to the homology of previous two bacteriocins with Plantazolicin and Pumilarin, respectively (Figure 3A). Additionally, we see that the organization of the genes comprising each putative C3 bacteriocin biosynthetic gene cluster are similar to the organization of their respective biosynthetic gene cluster homologs (Figure 3B).

### Structural Models of Bacteriocin-Like Proteins in C3 Unique Inserts

In order to better understand the two circular bacteriocins identified in C3, we sought to predict their secondary structures. Using BLAST, we determined that the C3 Safencin amino acid sequence shows a significant degree of conservation with AS-48 (Supplementary Figure 4). Particularly, conservation is greatest at the C-terminal end, including the residues involved in head-to-tail cyclization. To further investigate the structural similarity between these bacteriocins, we performed a structural model comparison using the known three-dimensional structure of the AS-48 bacteriocin (PDB ID 1O82). Three-dimensional modeling confirmed that Safencin adopts a conformational fold that is similar to the AS-48 bacteriocin from *E. faecalis* (Supplementary Figure 4).

Because the structure of Butyrivibriocin AR10 is unknown, we performed molecular modeling simulations of the Butyrivibriocin AR10 peptide sequence and the C3 homolog using the I-TASSER protein structure prediction program. Our simulation results revealed for the first time that Butyrivibriocin AR10, as well as the homologous product encoded by the C3 gene product adopts a fold that is similar to the AS-48 family of bacteriocins (Supplementary Figure 5). Our structural model of Butyrivibriocin AR10 demonstrates that multiple highly hydrophobic residues are not completely buried in the core of the cyclic peptide but are rather exposed on the peptide surface (Supplementary Figure 5; residues in orange and gray). This observation is consistent with the experimental data available for Butyrivibriocin AR10. Interestingly, *B. pumilus* B4107, which has a highly similar bacteriocin gene to C3 Safencin, does not contain a Butyrivibriocin AR10 homolog in its genome as analyzed by BAGEL4, indicating that these are likely distinct microbial isolates.

### Transcription of Genes Encoding Bacteriocins in *B. safensis*

**FIGURE 4:**
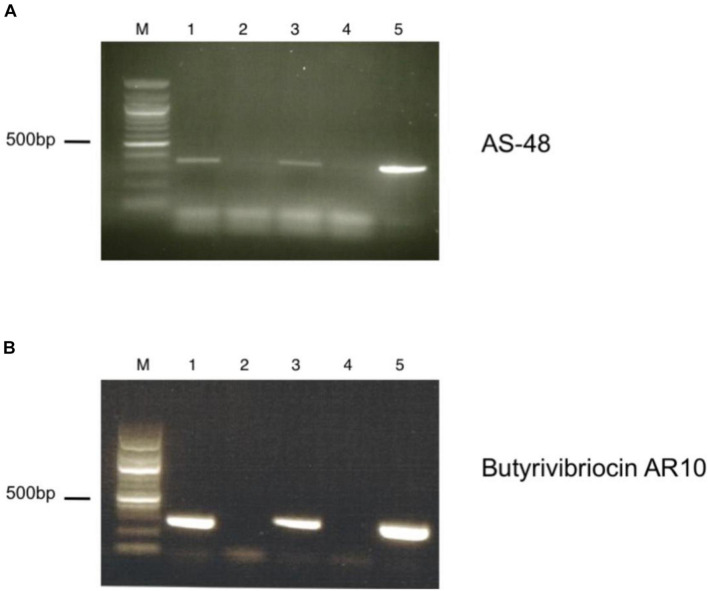
RT-PCR of putative bacteriocin-like genes present in the genome of C3. **(A)** Detection of AS-48 bacteriocin transcript by RT-PCR. Lanes are as indicated: M. Marker, (1) +RT (TH media), (2) -RT (TH media), (3) +RT (TH media supplemented with BSA), (4) -RT (TH media supplemented with BSA), (5) Positive DNA control. **(B)** Detection of Butyrivibriocin AR10 homolog bacteriocin transcript by RT-PCR. Lanes are as indicated: M. Marker, (1) +RT (TH media), (2) -RT (TH media), (3) +RT (TH media supplemented with BSA), (4) -RT (TH media supplemented with BSA), (5) Positive DNA control.

To verify the expression of the putative circular bacteriocin genes present in the genome of the C3 isolate, we performed reverse-transcriptase PCR using primers specific for the two circular bacteriocin open reading frames. We observed significant transcription of genes encoding both the Butyrivibriocin AR10 open reading frame and the AS-48 bacteriocin gene (Figure 4). These bacteriocins were observed to be transcribed during both log-phase and stationary phase cultures (data not shown). We did not observe PCR products in reverse-transcriptase omitted lanes, ensuring that PCR products were produced from cDNA and not due to genomic contamination of PCR template.

### Antimicrobial Peptide Purification

**FIGURE 5:**
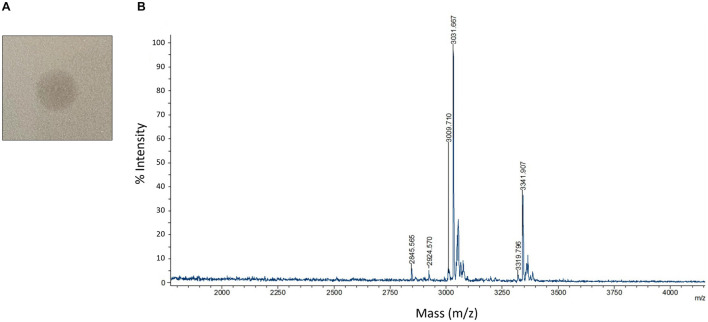
Detection and characterization of HPLC purified AMPs **(A)** HPLC purified AMP from C3 extracts was spotted on a confluent lawn of *B. subtilis* and displays a zone of growth inhibition. **(B)** MALDI-TOF detection of HPLC purified AMP digested by leucine aminopeptidase for 40 mins at 37°C. Peaks are labeled with *m/z* values.

To study the antimicrobial activities demonstrated by C3, we utilized protein extraction and purification methods including cation exchange chromatography (Supplementary Figure 6A) and HPLC (Supplementary Figure 6B) to isolate AMP candidates produced by C3. We successfully generated a high purity sample of peptide that, when plated on a confluent lawn of *B. subtilis*, demonstrated a clear zone of inhibition (Figure 5A). The peptide showed a m/z peak at ∼3,319.80 and 3,341.91, with the second value likely the result of a sodium adduct (+22) (Figure 5B). This mass did not correspond exactly to any of the three predicted bacteriocin expected masses as identified by BAGEL4.

Since we were unable to identify the antimicrobial product based solely on the mass to charge value of our product, we carried out a series of protease experiments to characterize the peptidic nature of the purified molecule. The isolated peptide was susceptible to digestion by Trypsin, proteinase K, and leucine aminopeptidase. Trypsin and proteinase K are both non-specific enzymes and therefore confirm general peptide properties of our active compound, with exposed amino acids susceptible to proteolysis. The capacity of leucine aminopeptidase to degrade the AMP indicates that this peptide is unmodified at the N-terminus. This is interesting since circular bacteriocins like Pumilarin and Butyrivibriocin AR10 have modifications to both termini, resulting from the cyclization of the peptide. Carboxypeptidase Y was unable to digest the peptide which may indicate the C-terminus is modified or capped. Endoproteinase GluC was also unable to digest the peptide, indicating that glutamic acid residues are not present in the peptide or these residues are resistant to digestion.

We next performed Edman degradation to identify the sequence of any possible amino acids present in the peptide. Edman analysis revealed two N-terminal amino acids for the first three positions as P1 W/S, P2 K/G, and P3 G/Q. These data are consistent with identification of the AS-48-like bacteriocin Safencin, as a result of the sequence SGG that was identified in our putative gene product (Figure 3A). Interestingly, the expected monoisotopic mass of a linear fragment of Safencin starting with SGG would be 3,326.95 Da, which is similar to what was observed with our MS analysis (Table 1). A difference of 7 Da was noted between a theoretical mass of the linear version of this bacteriocin, and the MS data, which likely presents an unknown loss or degradation of product prior or during MS. Our Edman analysis further indicates that the AMP may be a linear fragment or version of Safencin.

**TABLE 1:** MALDI-TOF *m/z* data of HPLC purified antimicrobial peptide (AMP) after leucine aminopeptidase digestion.

Linear sequence of C3 safencin peptide	Expected *m/z*	Observed *m/z*	Mass difference
SGGLSLIAAAGKETIRQYLKNEIKKKGRKAV	3,326.95	3,319.80	+7 Da
SLIAAAGKETIRQYLKNEIKKKGRKAV	3,012.79	3,009.71	+ 3 Da
LIAAAGKETIRQYLKNEIKKKGRKAV	2,925.76	2,924.57	+ 1 Da

We combined MALDI-TOF mass detection of the unknown peptide with a proteolytic digestion by leucine aminopeptidase to gain further information regarding the identity of the amino acid sequence composition of our purified peptide. During digestion, a major peak begins to form at 3,009.56 Da, a difference of 3 Da from the expected monoisotopic mass of Safencin (Table 1) subsequent to the digestion and removal of the N-terminal amino acids SGGL. We also observed a peak at 2,924.57 Da, which deviates from the expect monoisotopic mass of Safencin by only 1 Da. Given these findings, MS analysis identifies the active peptide as most likely a C-terminal fragment (residues 37–67) of the mature AS-48 Safencin peptide. While these data cannot confirm the entire sequence of all amino acids present in our sample, the data observed here shows similar patterns to what we would expect if our peptide were a novel linear peptide variant derived from Safencin, containing modifications that have not been identified.

### Artificial Colonization of *V. radiata* by C3

**FIGURE 6:**
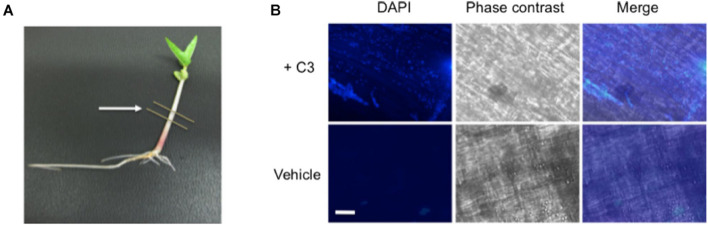
Microscopic images of C3 detection in bacteria inoculated seeds. **(A)** Following inoculation, *V. radiata* plants were placed directly under specific light conditions and grown for 7 days prior to processing, with young plants reaching an average height of 4–6 cm. Single plants were placed onto sterile plates for dissection. Lines indicate area removed for dissection and processing. Cuttings were directly placed onto glass slides and incubated with DAPI for visualization of bacteria. **(B)** 40× microscopic images show C3 bacilli colonized on plant cells (top panels). Plants treated with vehicle control show little bacteria compared with C3 inoculated samples. Plants remained healthy throughout the duration of the experiment. DAPI was used to visualize bacteria, and phase contrast merged images to locate the boundaries of the plant cell. White bar indicates an approximate measurement of 50 μm in length.

To confirm that our *B. safensis* C3 isolate was indeed capable of colonizing inside growing *V. radiata* seedlings, we established conditions to reintroduce C3 into imbibed seedlings and to assess the presence of C3 inside plant cells using microscopy. C3 was introduced to the seeds after they were surface-sterilized. Once the seeds germinated, *V. radiata* seedlings were grown for 7 days with young plants reaching an average height of 4–6 cm (Figure 6A). DAPI staining was used to identify bacteria within dissected plant stem tissue. Images showed the presence of C3 bacilli inside boundaries of plant cells (Figure 6B). In contrast, mock-inoculated plants showed little evidence of internal bacteria. Both C3-inoculated and mock-treated plants had similar rates of growth and appeared healthy for the duration of the experiment. These results indicate that inoculation of C3 into *V. radiata* during seed imbibition results in commensal states of bacterial species inside plant cells.

## Discussion

Our data provides strong evidence that the seeds of *V. radiata* can contain bacterial endospores that remain viable inside the seed. The isolation of endophytic microbes from surface-sterilized seeds has been reported previously in many other plants, including rice, corn, and cotton (Gond et al., 2015; Irizarry and White, 2017; Verma et al., 2017). In this study, we isolated a unique *B. safensis* strain, termed C3, from selected lots of *V. radiata* seeds. The C3 phenotypes observed (rapid growth on TH media, biofilm formation, and isolation from a surface-sterilized dry seed) indicate a close relationship between *V. radiata* and the *B. safensis* strain. The exceptional swarming behavior suggests that C3 was a natural isolate and not derived from laboratory-based sources (Barreto et al., 2020). Persistence in the dry environment (8–11% moisture by weight) within mung bean seeds (Murthy et al., 2003) would be possible for a *Bacillus*, given that viable endospores can be found within rocks (Fajardo-Cavazos and Nicholson, 2006). Other *Firmicutes* can persist in environments as arid as the Atacama desert, the driest place on earth (Drees et al., 2006).

We have shown that C3 is taken up during imbibition, propagates within the plant body and can be reisolated from the mung bean seed. The seeds of *V. radiata*, as well as those of other herbaceous, annual Faboideae (e.g., beans, chickpeas, and soybeans) have been shown to mature inside of closed pods. The young pods of *Phaseolus vulgaris* (common bean) contain stomata on the outer surface but these are partially to fully obstructed (Crookston et al., 1974). Cells in the inner half of the pod contain no chloroplasts and do not form stoma. The pods of *V. radiata* are likely to be similar. If the *Bacillus* can persist within the plant body, differentiating floral tissues may transmit the bacteria through the pollen or through the megagametophyte.

Previous investigations have shown that *B. safensis*, a naturally occurring soil microbe, is associated with the plant rhizosphere and promotes plant growth (Sarkar et al., 2018). Protection from pathogens may contribute to the observed positive effects on host plant growth. Living *B. safensis* C3, applied to vegetables preharvest could inhibit or even prevent bacterial colonization by human pathogens. Here we described three bacteriocins encoded in the genome of C3, two circular bacteriocins, Safencin, and Butyrivibriocin AR10-like, and one LAP, we refer to as Plantazolicin-like. These circular bacteriocins have the unique feature of N- and C- terminal peptide bond linkages (Maqueda et al., 2008). To date there are 15 circular bacteriocins described in the literature with AS-48 being the best studied. Recent *in silico* analysis of 6,928 known and putative circular bacteriocins precursor peptides classifies Pumilarin with AS-48 based on high amino acid sequence similarity (Xin et al., 2020). Our structural modeling of Safencin, which only differs from Pumilarin by one amino acid, suggests that this sequence similarity may also extend to three-dimensional structure. Safencin appears to preserve the spatial orientation of specific residues which are critical for the transition of AS-48 water-soluble form to a membrane-bound state upon membrane binding (Sánchez-Barrena et al., 2003). Interestingly, circular bacteriocins have physiochemical features that are often shared by the theta-defensins, mammalian AMPs produced by the innate immune system for combating bacterial infection (Selsted, 2005).

Edman degradation data that we obtained is a strong indication that the peptide that we isolated is a linear form that is derived from the AS-48-like bacteriocin gene. While we were able to confirm the production of an AMP from C3 that is derived from a circular AS-48-like bacteriocin, we were not able to determine the exact molecular structure of the form. Using multiple analytical methods, we identified a partial sequence that directly corresponded with the AS-48-like biosynthetic gene product, as annotated in the C3 genome. Resistance to digestion by carboxypeptidase Y may be due to a possible C-terminal modification or other posttranslational modification of the peptide that heretofore has not been identified to occur in these bacteriocin families. Although circular bacteriocins are resistant to proteolysis (Gabrielsen et al., 2014), our results show that there may be a linear form of the circular peptide present in the bacterial cultures that still retains significant antimicrobial activity.

While we know of several circular bacteriocins have been reported in the literature, no natural linear variants of the AS-48 bacteriocin have been isolated from natural sources (Xin et al., 2020). Previous work has shown that AS-48-like bacteriocins can still retain antimicrobial activity when reduced to a linear version (Montalbán-López et al., 2008; Fields et al., 2018; Ross et al., 2020). The findings with Safencin demonstrated that only the cationic C-terminal residues 39–70 of the mature peptide were required for antimicrobial activity (Fields et al., 2018). This is the first evidence that a circular bacteriocin can be processed to a natural linearized form by the microorganism to retain antimicrobial activity. We cannot rule out the possibility that the mature Safencin peptide is exported conventionally by the producing organism as a circular peptide, which is then proteolytically processed by either *B. safensis* or by the *B. subtilis* which is present in these co-cultures.

We have demonstrated the genetic capability of a *B. safensis* strain to produce three distinct bacteriocins. Taken altogether, our work suggests that C3 serves as a commensal species of *V. radiata* and that C3 bacteriocins inhibit the growth of plant pathogens and bacterial strains that may be pathogenic to humans. As bacteriocins are likely to be safe for human consumption, we are currently investigating the activity of these and other novel bacteriocins against pathogens of interest.

Future research including experiments to gain more detailed information regarding the possible linear structure of the AMP we purified from C3, along with genetic studies to identify possible biosynthetic mechanisms involved in producing linear forms of circularized bacteriocins are planned. Nonetheless, our data reveal that bacteriocin-producing commensal microbes in the plant community may serve as an important component of the overall microbiome. The recent recognition of the complexity of the naturally occurring plant microbiome and the potential utility of this microbiome for the protection of both plant and produce opens a whole new area of systems biology that could have a significant impact on human health and sustainable food production.

## Data Availability Statement

The datasets presented in this study can be found in online repositories. The names of the repository/repositories and accession number(s) can be found below: NCBI GenBank, accession no: JAIFAE000000000.

## Author Contributions

SL, TM, and JR-S designed and conceived the experiments. TM, DS, FF, SP-J, CT, EP, JS, and MP conducted the studies and obtained the data. JS, TM, and SL prepared the manuscript with assistance from other listed co-authors. All authors contributed to the article and approved the submitted version.

## Conflict of Interest

The authors declare that the research was conducted in the absence of any commercial or financial relationships that could be construed as a potential conflict of interest.

## Publisher’s Note

All claims expressed in this article are solely those of the authors and do not necessarily represent those of their affiliated organizations, or those of the publisher, the editors and the reviewers. Any product that may be evaluated in this article, or claim that may be made by its manufacturer, is not guaranteed or endorsed by the publisher.
