# Supplementary material for: A Seed-Endophytic Bacillus safensis Strain With Antimicrobial Activity Has Genes for Novel Bacteriocin-Like Antimicrobial Peptides
Source: Front Microbiol. 2021 Sep 27;12:734216. doi: 10.3389/fmicb.2021.734216 (PMC8503640; doi:10.3389/fmicb.2021.734216)
Supplement: Supplementary file 1 [file Data_Sheet_1.docx]

Supplementary Material

# Supplementary Figures


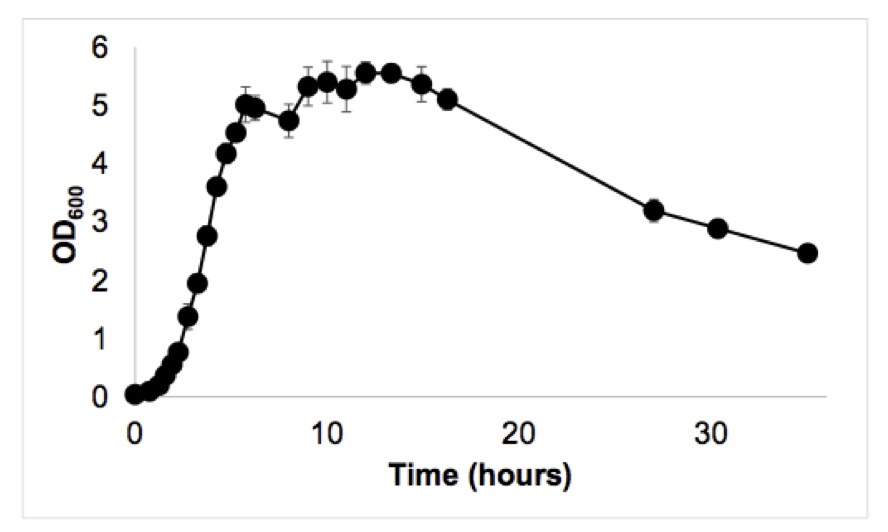


**Supplementary Figure 1.** Growth curve of bacterial isolate C3. Bacteria were grown in LB for the timepoints indicated. OD600 was measured as indication of bacterial growth.

**
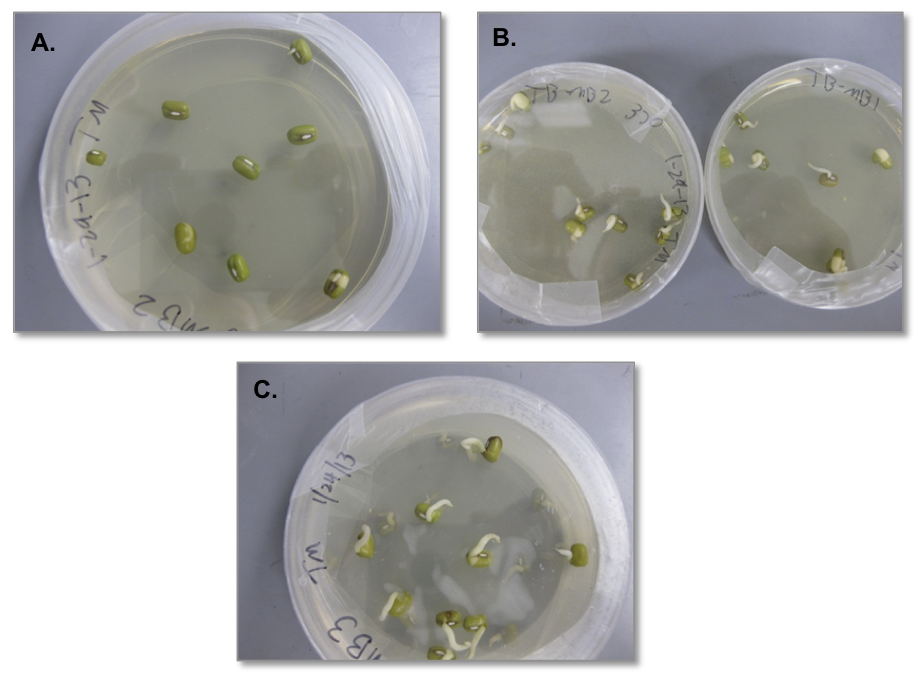
**

**Supplementary Figure 2**. *Vigna radiata* seeds grown in nutrient agar containing LB broth show absence of contaminating surface-laden bacteria. Images indicate seeds incubated in agar plates. Bacterial growth was not detected in any of the tested conditions. Plates were incubated for the duration and temperature as follows: A. Seeds grown in LB plates containing 1% agar for 3 days at 20°C; B. Seeds grown in LB plates (1% agar) for 3 days at 37°C; C. Seeds grown in LB plates (1.5% agar) for 7 days at 20°C.


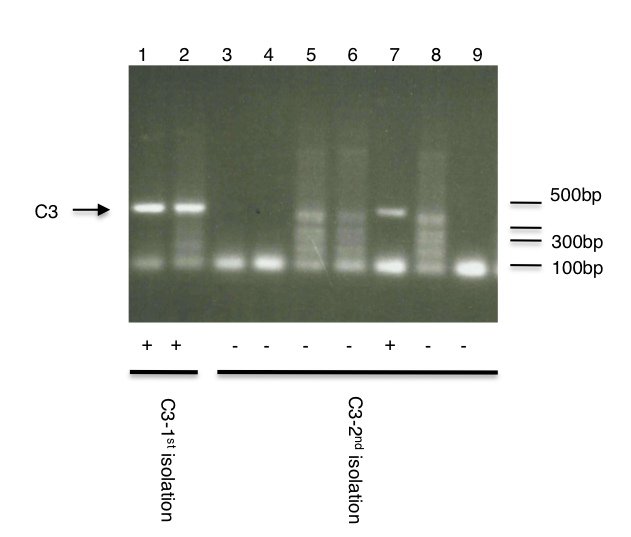


**Supplementary Figure 3.** PCR Detection of *B. safensis* C3 from multiple seed extracts of *Vigna radiata*. Primers were designed to amplify a 400bp unique region of C3. Lanes 1 and 2 represent colony PCR products from the original isolate of C3. Lanes 3 through 9 depict colony PCR products from an independent extraction of seed products. Reisolation of C3 is confirmed by positive colony PCR of C3-specific product visible in lane 7.


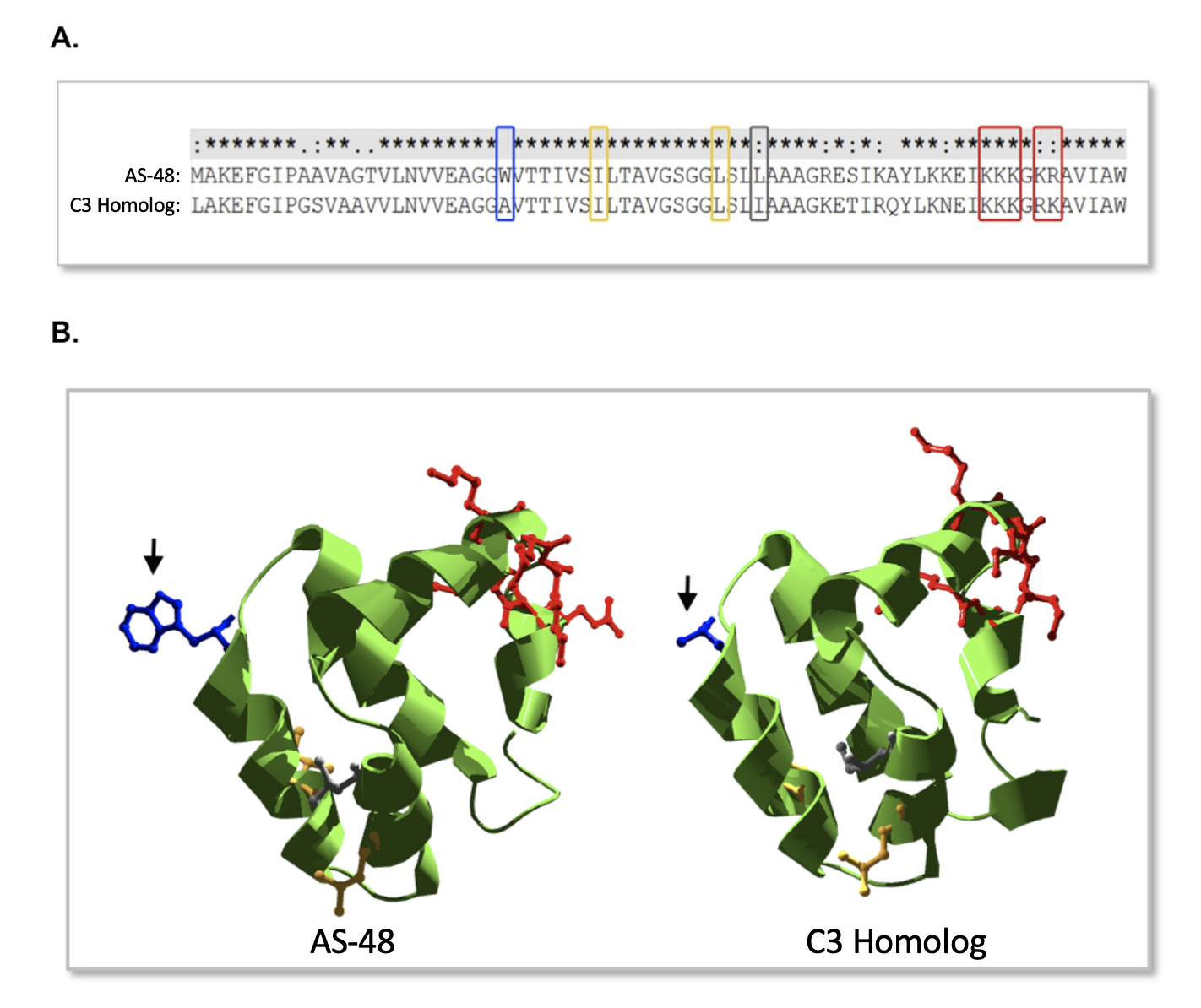


**Supplementary Figure 4.** Structural comparison of the AS-48 bacteriocin from *E. faecalis* to the Pumilarin-like homolog encoded by the C3 gene. A. Sequence alignment of AS-48 and homolog in C3. The alignment includes the residues forming the cyclic mature form of AS-48 (residues 36-105), and their equivalents in the newly discovered bacteriocin (residues 39-108), respectively. Stars (*) denotes identical residues. Columns (:) denote conserved residues. Single dots (.) denote semi-conserved residues. The amino acids forming the cluster of charged residues (see text) are denoted with red rectangles. The amino acids forming the cluster of surface exposed hydrophobic residues (see text) are denoted with orange (identical for both sequences), and gray (conserved in both sequences) rectangles. The non-conserved Trp residue is denoted with blue rectangle. The alignment was performed with ClustalX2 software. B. Tertiary structure comparison of AS-48 and the Pumilarin-like bacteriocin from C3. The structure model of the new homolog was created by ModWeb Comparative Modeling Server available via the Protein Model Portal. The amino acids forming the cluster of charged residues (see text) are shown in red. The amino acids forming the cluster of surface exposed hydrophobic residues are shown in orange (identical residues), and gray (conserved residues). The non-conserved Trp residue and its Ala analog are shown in blue. The structures were visualized by SPDBV software.


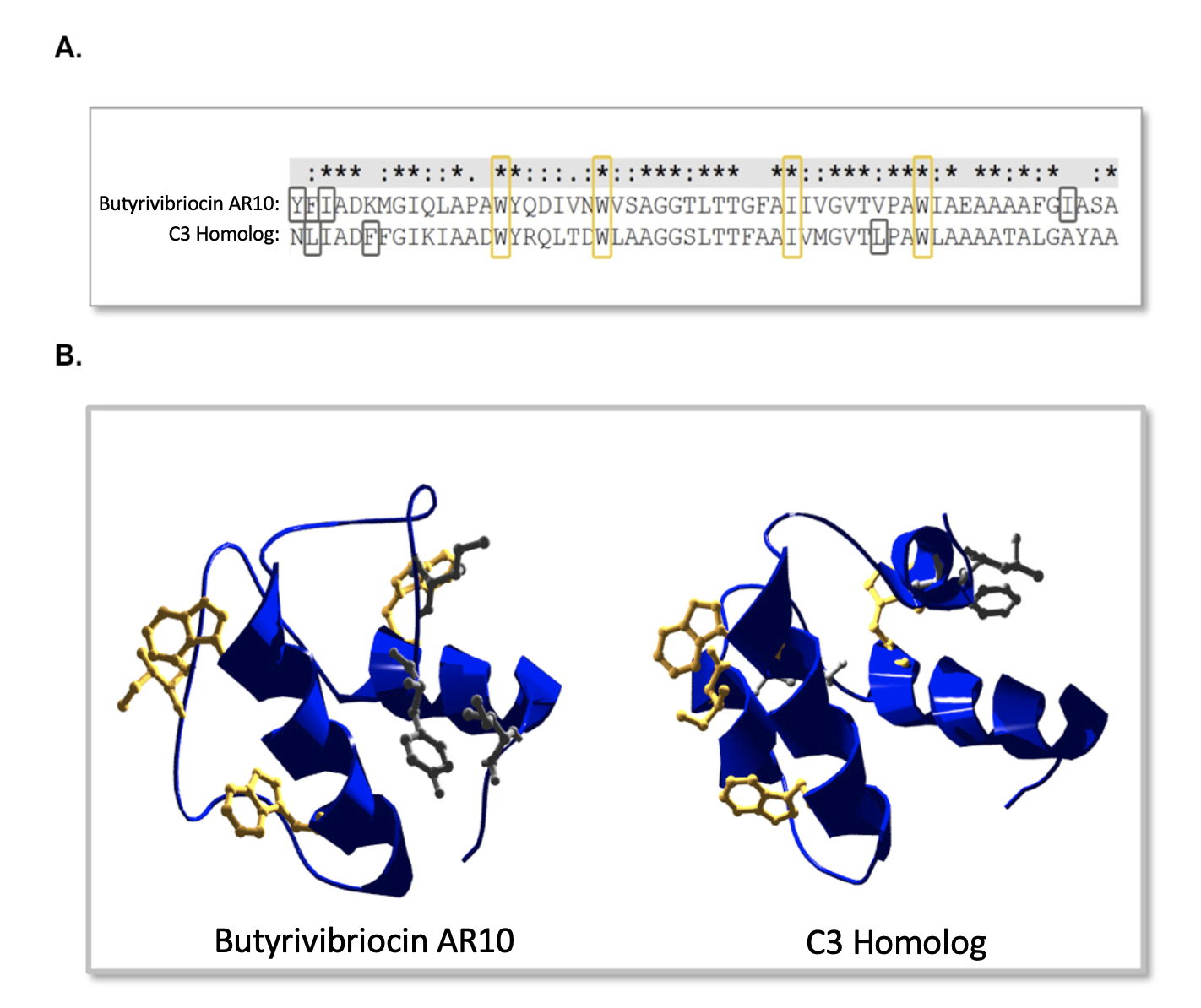


**Supplementary Figure 5.** Structural analysis of the Butyrivibriocin AR10 homolog found in C3. A. Sequence alignment of Butyrivibriocin AR10 and its newly discovered homolog. The alignment includes the residues forming the cyclic mature form of Butyrivibriocin AR10 (residues 24-85), and their equivalents in the newly discovered bacteriocin (residues 37-93), respectively. Stars (*) denotes identical residues. Columns (:) denote conserved residues. Single dots (.) denote semi-conserved residues. Hydrophobic residues which are partially or completely exposed on the surface of the structure model are denoted with orange (identical for both sequences), and gray (conserved in both sequences) rectangles. The alignment was performed with ClustalX2 software. B. Structure models of Butyrivibriocin AR10 and its new homolog. The structure models were created by I-TASSER protein structure prediction program. The Confidence scores (C-scores) for Butyrivibriocin AR10 and its homolog are -1.56 and -1.13, respectively (typical C-score values range is -5 to 2, with higher values correlating with higher confidence). Hydrophobic residues which are partially or completely exposed on the surface of the structure model are shown in orange (identical residues), and gray (conserved residues). The structures were visualized by SPDBV software.

**A.
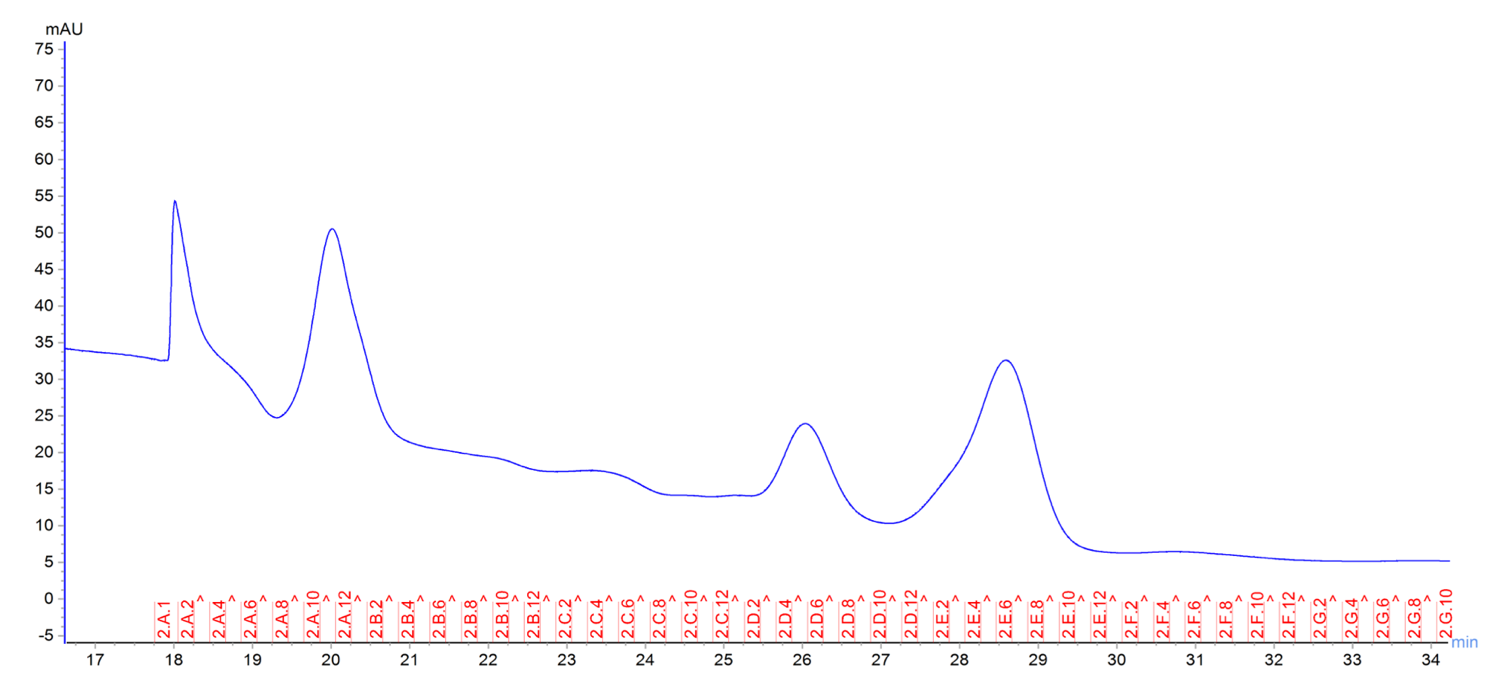
**

**B.**

**
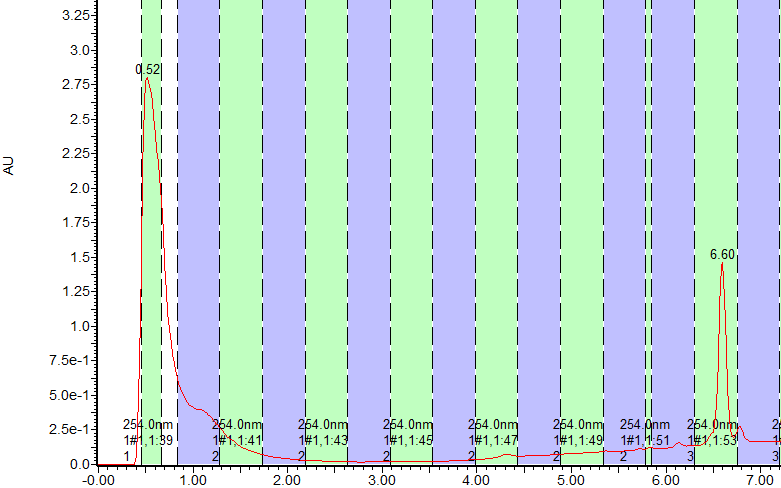
**

Absorption units (AU)

Time (min)

**Supplementary Figure 6.** Detection of antimicrobial peptide fractions during protein purification steps. A. Cation exchange chromatography elution step shows multiple peaks. Peak #2 (blue arrow) showed antimicrobial activity using the spot-on-lawn technique. B. Reversed-phase HPLC chromatogram shows two peaks. The second peak showed antimicrobial activity when detecting activity using spot-on-lawn technique and had a retention time of 6.60 mins.
